# HGF-Modified Dental Pulp Stem Cells Mitigate the Inflammatory and Fibrotic Responses in Paraquat-Induced Acute Respiratory Distress Syndrome

**DOI:** 10.1155/2021/6662831

**Published:** 2021-03-02

**Authors:** Panpan Geng, Yuning Zhang, Huan Zhang, Xiwen Dong, Yuefeng Yang, XiaoNa Zhu, Chu-Tse Wu, Hua Wang

**Affiliations:** ^1^Department of Experimental Hematology, Beijing Institute of Radiation Medicine, Beijing 100850, China; ^2^The Fifth Department of Chemotherapy, Affiliated Tumor Hospital of Guangxi Medical University, Nanning 530021, China; ^3^Beijing SH Biotech Corporation, Beijing 100070, China; ^4^Beijing Key Laboratory for Radiobiology, Beijing 100850, China

## Abstract

Paraquat (PQ) poisoning can cause acute lung injury and progress to pulmonary fibrosis and eventually death without effective therapy. Mesenchymal stem cells (MSCs) and hepatocyte growth factor (HGF) have been shown to partially reverse this damage. MSCs can be derived from bone marrow (BM-MSCs), adipose tissue (AD-MSCs), umbilical cord (UC-MSCs), dental pulp (DPSCs), and other sources. The biological characteristics of MSCs are specific to the tissue source. To develop an effective treatment for PQ poisoning, we compared the anti-inflammatory and antifibrotic effects of UC-MSCs and DPSCs and chose and modified a suitable source with HGF to investigate their therapeutic effects in vitro and in vivo. In this study, MSCs' supernatant was beneficial to the viability and proliferation of human lung epithelial cell BEAS-2B. Inflammatory and fibrosis-related cytokines were analyzed by real-time PCR. The results showed that MSCs' supernatant could suppress the expression of proinflammatory and profibrotic cytokines and increase the expression of anti-inflammatory and antifibrotic cytokines in BEAS-2B cells and human pulmonary fibroblast MRC-5. Extracellular vesicles (EVs) derived from MSCs performed more effectively than MSCs' supernatant. The effect of DPSCs was stronger than that of UC-MSCs and was further strengthened by HGF modification. PQ-poisoned mice were established, and UC-MSCs, DPSCs, and DPSCs-HGF were administered. Histopathological assessments revealed that DPSCs-HGF mitigated lung inflammation and collagen accumulation more effectively than the other treatments. DPSCs-HGF reduced lung permeability and increased the survival rate of PQ mice from 20% to 50%. Taken together, these results indicated that DPSCs can suppress inflammation and fibrosis in human lung cells better than UC-MSCs. The anti-inflammatory and antifibrotic effects were significantly enhanced by HGF modification. DPSCs-HGF ameliorated pulmonitis and pulmonary fibrosis in PQ mice, effectively improving the survival rate, which might be mediated by paracrine mechanisms. The results suggested that DPSCs-HGF transplantation was a potential therapeutic approach for PQ poisoning.

## 1. Introduction

Paraquat (1,1′-dimethyl-4,4′-bipyridinium dichloride, PQ) is a widely used herbicide, especially in developing countries, due to its strong oxidative and nonselective properties, low cost, and inexpensive manufacturing process [1, 2]. However, the incidence of oral or accidental ingestion of PQ has increased dramatically in the past two decades. PQ poisoning causes injury to multiple organs, such as the liver, kidney, heart, and central nervous system, but the lung is the primary target tissue [3, 4]. The mortality of PQ poisoning is as high as 90%, with no effective antidote or pharmacological treatment options other than minimizing its absorption and attempting to prevent tissue injury [1, 5, 6]. Although PQ use has been prohibited in many countries, it is still the main component of other herbicides, such as Diquat in China. Therefore, it is necessary to develop an effective clinical treatment strategy.

At present, it is generally believed that PQ initially leads to acute respiratory distress syndrome (ARDS), including pulmonary edema and hemorrhage, and the infiltration of inflammatory cells into the interstitium and alveoli in the early stage [1, 3]. With the progression of the disease, pulmonary fibrosis and pulmonary interstitial fibrosis occur, and respiratory failure is the main cause of death from PQ poisoning [7]. Therefore, regulating and minimizing the progression of inflammation and fibrosis may be an effective strategy for treating PQ poisoning.

Recently, mesenchymal stem cells (MSCs) have been used to treat lung injury because of their low immunogenicity and paracrine effects [8, 9]. MSCs can be derived from various sources, including bone marrow, umbilical cord, dental pulp, adipose tissue, and placenta [8]. Bone marrow-derived MSCs (BM-MSCs) and adipose-derived MSCs (AD-MSCs) require invasive surgery, such as puncture or liposuction, which increases the risk of infection. Umbilical cord and teeth are medical waste, and MSCs obtained from the umbilical cord (UC-MSCs) and dental pulp (DPSCs) cause nearly no damage to the donors. In addition, these cells have strong proliferation abilities and low immunogenicity and are thus widely used. Moreover, the biological properties of MSCs may vary between cells from different sources because of tissue specificity and plasticity [10–12]. Thus, the selection of a suitable source of MSCs for transplantation is important for the treatment of PQ poisoning in clinical medicine.

Hepatocyte growth factor (HGF) is a multifunctional cytokine that prevents and attenuates disease progression by influencing multiple pathophysiological processes involved in inflammatory and immune response, including cell migration, maturation, cytokine production, antigen presentation, and T cell effector function [13]. Moreover, HGF was reported to have the function of suppressing lung fibrosis. Several studies approved that HGF could alleviate an injured lung of ARDS via protection lung permeability. For example, HGF protected vascular endothelial barrier dysfunction and apoptosis [14]; Hu et al. found that HGF-overexpressed MSCs maintained lung permeability and protected the lung injury induced by LPS [15]. The symptom of PQ poisoning in the lung is the lung toxicities including pulmonary hemorrhage, congestion, edema, and fibrosis [3]. It is similar to the pathological features of ARDS which is characterized by diffuse injury to the lung endothelial and epithelial cells, increased alveolar-capillary permeability, and alveolar pulmonary edema. Besides the lung permeability and alveolar pulmonary edema, the histopathology of PQ poisoning also includes inflammatory infiltration and rapid development of fibrosis, so it would be beneficial if the treatment could suppress the fibrosis response. HGF-induced inhibition of fibrotic remodeling may occur via multiple direct and indirect mechanisms including the promotion of cell survival and proliferation of pulmonary epithelial and endothelial cells and the reduction of myofibroblast accumulation [16]. Watanabe et al. reported that HGF was identified as a key ligand to elicit myofibroblast apoptosis and extracellular matrix degradation in increasing activities of matrix metalloproteinases- (MMP-) 2/9 [17]. The abovementioned studies illustrated that HGF could regulate inflammation and pulmonary fibrosis. So, we modified the most promising MSCs with the HGF gene to treat PQ poisoning in this study.

In addition, the biologically active components secreted by MSCs are not only cytokines but also extracellular vesicles (EVs), which are rich in proteins, mRNAs, miRNAs, and other substances that participate in cell-to-cell communication [18, 19]. EVs can alleviate the development of pulmonary fibrosis [20].

In this study, we cultured human lung epithelial cells (BEAS-2B) and human pulmonary fibroblasts (MRC-5) with the supernatant of UC-MSCs or DPSCs to compare the anti-inflammatory and antifibrotic effects of these two types of MSCs, and the effects of MSCs-derived EVs were investigated simultaneously. Then, the optimal source of MSCs was selected and modified with HGF, and the inhibitory effect of these modified cells on inflammation and fibrosis was analyzed to investigate the therapeutic effect on PQ-induced lung injury. These results provide the first comprehensive evaluation of MSCs transplantation for the treatment of PQ poisoning.

## 2. Materials and Methods

### 2.1. Cells

Human UC-MSCs and DPSCs were gifts from Beijing SH Biotech Corporation. The two types of MSCs were used at passages 4-6 and were cultured with serum-free media (SANYL, Beijing Sanyouli Technology Advanced Co., China). All the experimental procedures were approved by the institute ethics committee.

Human lung epithelial cells (BEAS-2B) were kindly gifted by Professor Zhou (Beijing Key Laboratory for Radiobiology) and cultured in a growth factor and hormone-supplemented medium (LHC-8) (Gibco BRL, Grand Island, NY, USA). Normal adult human lung fibroblasts (MRC-5) were obtained from ATCC and were cultured in DMEM supplemented with 10% fetal bovine serum and 1% NEAA (Gibco BRL, Grand Island, NY, USA). MSCs-derived culture supernatant and EVs were collected or isolated for coculture with BEAS-2B and MRC-5 cells.

### 2.2. Adenoviral Vectors and Gene Modification

The adenoviruses (AdVs) used in this study were Ad-HGF and Ad-null. AdVs are replication-defective adenoviruses. Ad-HGF expresses human HGF, and Ad-null does not carry exogenous genes. Both vectors were constructed with the AdEasy system (Stratagene, La Jolla, CA, USA) as previously described [21]. DPSCs were infected at a multiplicity of infection (MOI) of 150 with Ad-HGF or Ad-null, and the supernatant or cells were collected 2 days postinfection.

### 2.3. Cell Viability and Proliferation Assay

Cell Counting Kit-8 (CCK8) (Bimake, Shanghai, China) was used to measure the cell viability at 24 h, 48 h, and 72 h after culture according to the manufacturer's instructions. BEAS-2B were seeded in 96-well plates (2 × 10^3^ cells per well) with 10 *μ*L/well MSCs' supernatant. The control group was cultured with an equal volume of fresh medium.

BEAS-2B were seeded in 6-well plates (2 × 10^4^ cells per well) with 200 *μ*L/well MSCs' supernatant for 72 h. The proliferation index was determined by flow cytometry with the cell proliferation dye eFluor® 670 (Dye 670) (Invitrogen, Carlsbad, CA, USA) according to the manufacturer's instructions.

### 2.4. Lung Cell Inflammation and Fibrosis Induction

BEAS-2B and MRC-5 cells were seeded separately at 2 × 10^5^ cells per well in 6-well cell culture plates (Nalge Nunc International, Rochester, NY, USA). TGF-*β* (PeproTech, NJ, USA) was used at a final concentration of 10 ng/mL for 48 h to induce inflammation and fibrosis in vitro. Then, BEAS-2B or MRC-5 cells were cultured with 200 *μ*L/well MSCs' supernatant or approximately 1.8 × 10^4^ EVs/cell for 48 h according to our previous study [22].

### 2.5. RNA Isolation and Reverse Transcription-Polymerase Chain Reaction

Total RNA was isolated from cells using the RNA-Quick Purification Kit (ES Science, Shanghai, China). Total RNA was isolated from lung tissue using the TRIzol Reagent (Sigma-Aldrich, St. Louis, MO, USA). cDNA was synthesized using a StarScript II First-strand cDNA Synthesis Mix with a gDNA Remover kit (GenStar, Beijing, China). Quantitative polymerase chain reaction (qPCR) was performed using the RealStar Green Fast Mixture with ROX II (GenStar, Beijing, China) on an ABI 7500 fast system (Applied Biosystems, Thermo Fisher Scientific, CA, USA). PCR analyses were performed in quadruplicate for each sample, and the mRNA levels were measured using a standard curve. Inflammation-related and fibrosis-related factors were analyzed in this study. The proinflammatory cytokines included TNF-*α*, IL-1*β*, IL-6, and IL-8, and the anti-inflammatory cytokines included IL-4, IL-10, and IL-13. The fibrosis-related factors included *α*-SMA, vimentin, collagen I, tissue inhibitor of matrix metalloproteinases (TIMP), matrix metalloproteinase- (MMP-) 9, and E-cadherin. The primer sequences are shown in detail in Table 1.

### 2.6. PQ Model and MSCs Transplantation Assays

All the animal experiments were approved by the Ethics Committee of Beijing Institute of Radiation Medicine, and all the procedures were conducted in accordance with relevant guidelines and regulations. C57BL/6J mice (female, SPF grade) were purchased from Beijing Vital River Laboratory Animal Technology Co., Ltd. (Beijing, China). Forty-five mice were randomly divided into 5 groups: normal group (*n* = 5), PQ group (*n* = 10), UC-MSCs group (*n* = 10), DPSCs group (*n* = 10), and DPSCs-HGF group (*n* = 10). According to the preliminary experimental results, the mice were intragastrically administered 0.2 mL of PQ (Sigma-Aldrich, St. Louis, MO, USA) at a dose of 150 mg/kg, and the normal group was gavaged with the same volume of saline.

UC-MSCs, DPSCs, or DPSCs-HGF were injected into PQ-poisoned mice via the tail vein in 200 *μ*L of saline (1 × 10^6^ cells) on days 1 and 3 after intragastric PQ administration. The PQ group received 200 *μ*L of saline without any cells. Deaths were recorded for each group. The remaining mice were sacrificed on day 28.

### 2.7. Histopathological Assessment

The right lung lobes were fixed in 4% paraformaldehyde (Servicebio Technology, Wuhan, Hubei). Hematoxylin and eosin (H&E) staining was performed to assess tissue inflammation and morphology, and Masson staining was performed to quantify tissue fibrosis.

Photomicrographs at magnifications of ×400 were obtained using a light microscope (Olympus CKX53; Olympus Latin America Inc., Brazil). The inflammation injury degree was graded using a previously described scoring system with slight modifications [23]. The pathological features including inflammatory cell infiltration, hemorrhage, and type II alveolar epithelial hyperplasia are graded from 0 to 4 based on its absence (0) or presence to a slight (1), mild (2), moderate (3), or severe (4) degree. Ten microscopic fields were randomly selected from each Masson-stained section to evaluate the tissue fibrosis, and the percentage of the collagen fiber pixel area in the total tissue pixel area was statistically determined.

### 2.8. Enzyme-Linked Immunosorbent Assay

Bronchoalveolar lavage fluid (BALF) was collected and centrifuged at 4°C with 8000 rpm for 10 minutes. Supernatants were stored at −70°C for protein and cytokine analysis. Albumin (ALB), alkaline phosphatase (ALP), and hydroxyproline (HYP) in BALF supernatants were quantified using commercially available enzyme-linked immunoabsorbent assays (MLbio, Shanghai, China) according to the manufacturer's instructions.

### 2.9. Statistical Analysis

The data are expressed as the mean ± SEM or with GraphPad Prism 6 (GraphPad Software, San Diego, CA, USA). Comparisons between groups were measured by ANOVA with Tukey's or Dunn's multiple comparisons test to evaluate the statistical significance. *P* value ≤ 0.05 was considered statistically significant.

## 3. Results

### 3.1. MSCs Promoted the Viability and Proliferation of BEAS-2B Cells

**Figure 1:**
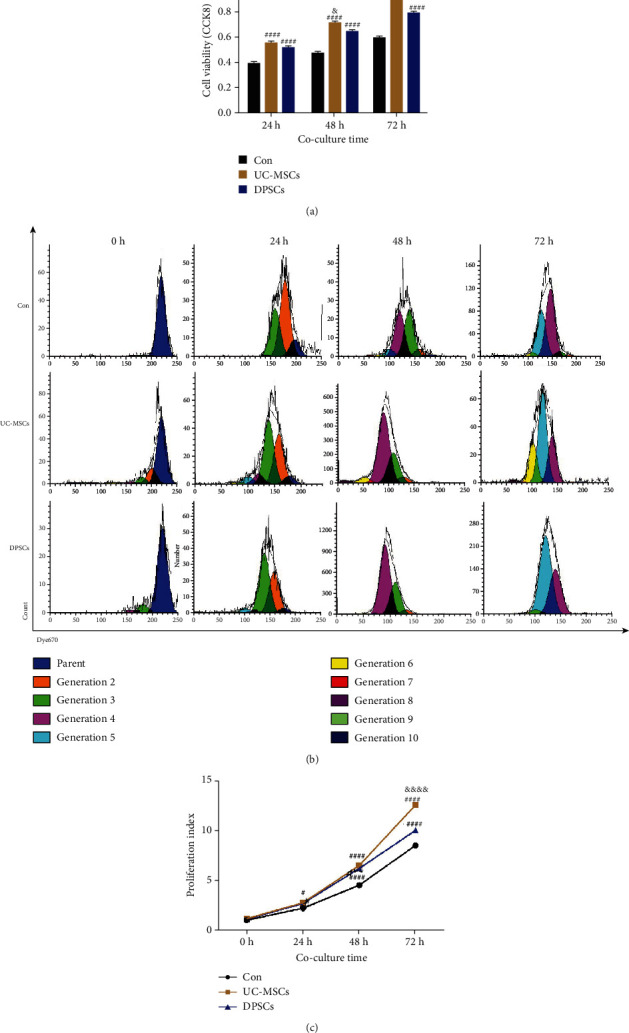
MSCs promote the viability and proliferation of BEAS-2B cells. (a) Cell viability was measured by CCK8 assays at 24 h, 48 h, and 72 h. *N* = 4. (b) Flow cytometric analysis of BEAS-2B proliferation after culture with fresh medium (top) or the supernatant of UC-MSCs (middle) or DPSCs (bottom) and the fluorescence intensity of Dye 670 at 0 h, 24 h, 48 h, and 72 h after staining. (c) Statistical analysis of the control and MSCs groups. *N* = 3. ^#^*P* < 0.01 and ^####^*P* < 0.0001 vs. Con; ^&^*P* < 0.05, ^&&&^*P* < 0.001, and ^&&&&^*P* < 0.0001 vs. the DPSCs group. Data are shown as the means ± SEM, and comparisons between groups were measured by ANOVA followed by Tukey's multiple comparisons test. Con: control; UC-MSCs: umbilical cord mesenchymal stem cells; DPSCs: dental pulp mesenchymal stem cells.

BEAS-2B cells were cultured with the supernatant of UC-MSCs and DPSCs separately for 3 days, cell viability was measured by CCK8 assays, and proliferation was analyzed by flow cytometry after the cells were stained with Dye 670. The CCK8 assay results demonstrated that the two MSCs treatment groups had higher viabilities than the control group, and the UC-MSCs group had higher cell viability than the DPSCs group (Figure 1(a)). The flow cytometry results also showed that the two MSCs groups had higher proliferation indexes than the control group, and the UC-MSCs group exhibited better proliferation indexes than the DPSCs group (Figures 1(b) and 1(c)). These results indicated that MSCs could promote the viability and proliferation of BEAS-2B cells and that UC-MSCs performed slightly better than DPSCs.

### 3.2. Anti-inflammatory Effects of UC-MSCs and DPSCs on BEAS-2B and MRC-5 Cells

**Figure 2:**
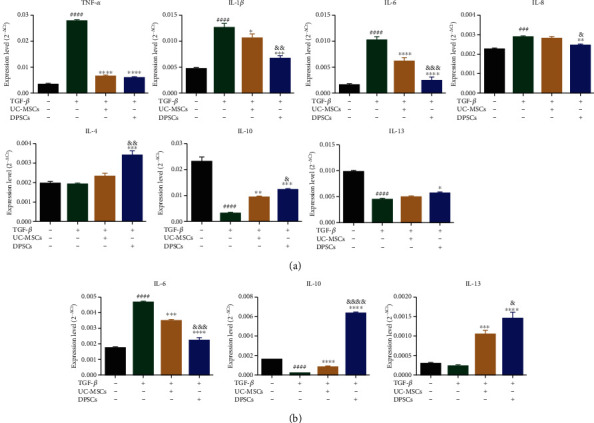
DPSCs alleviate the inflammatory responses of BEAS-2B and MRC-5 cells better than UC-MSCs. (a) The expression of inflammatory cytokines, including TNF-*α*, IL-1*β*, IL-6, IL-8, IL-4, IL-10, and IL-13, in BEAS-2B cells was evaluated by qPCR. (b) The expression of inflammatory cytokines, including IL-6, IL-10, and IL-13, in MRC-5 cells was measured by qPCR. Each experiment was performed in triplicate. The data are shown as the means ± SEM. ANOVA followed by Tukey's multiple comparisons test was used to evaluate these data. ^###^*P* < 0.001 and ^####^*P* < 0.0001 vs. Con; ^∗^*P* < 0.05, ^∗∗^*P* < 0.01, ^∗∗∗^*P* < 0.001, and ^∗∗∗∗^*P* < 0.0001 vs. the TGF-*β* group; ^&^*P* < 0.05, ^&&^*P* < 0.01, ^&&&^*P* < 0.001, and ^&&&&^*P* < 0.0001 vs. UC-MSCs. Con: control; UC-MSCs: umbilical cord mesenchymal stem cells; DPSCs: dental pulp mesenchymal stem cells.

To elucidate the anti-inflammatory effects of UC-MSCs and DPSCs on BEAS-2B and MRC-5 cells after TGF-*β* treatment, we measured several proinflammatory and anti-inflammatory cytokines by qPCR. The proinflammatory cytokines included tumor necrosis factor- (TNF-) *α*, interleukin- (IL-) 1*β*, IL-6, and IL-8, and the anti-inflammatory cytokines included IL-4, IL-10, and IL-13. In BEAS-2B cells, proinflammatory cytokines were decreased and anti-inflammatory cytokines were increased in the two MSCs groups compared to the TGF-*β* group (Figure 2(a)). Among these cytokines, TNF-*α* decreased dramatically and was nearly normal. The DPSCs group had notably decreased IL-1*β*, IL-6, and IL-8 compared with the UC-MSCs group, and there were significant differences between the two groups. There was no statistically significant difference in the expression levels of IL-8 and the anti-inflammatory cytokines IL-4 and IL-13 between the UC-MSCs group and the TGF-*β* group. However, the DPSCs group had significantly increased expression of anti-inflammatory cytokines, especially IL-4 and IL-10. The results indicated that UC-MSCs and DPSCs could inhibit proinflammatory cytokine release from BEAS-2B cells stimulated by TGF-*β*, and the effect of DPSCs was stronger than that of UC-MSCs. Moreover, DPSCs could induce BEAS-2B cells to secrete anti-inflammatory cytokines better than that of UC-MSCs. BEAS-2B cells were then cultured with DPSCs-EVs to investigate the anti-inflammatory effects of EVs. The results showed that DPSCs-EVs downregulated IL-6 and upregulated IL-4 more strongly than DPSCs (Supplementary Figure 1).

To determine whether the anti-inflammatory effect of MSCs on MRC-5 cells was consistent with that of BEAS-2B cells, we also measured the expression of IL-6, IL-10, and IL-13 in MRC-5 cells (Figure 2(b)). In MRC-5 cells, the two MSCs groups exhibited decreased IL-6 and increased IL-10 and IL-13 expression, similar to the effects on BEAS-2B cells. Furthermore, the DPSCs group had statistically significant differences when compared to the UC-MSCs group. These results indicated that both types of MSCs could inhibit proinflammatory cytokines and promote anti-inflammatory cytokine expression, and DPSCs may alleviate the inflammatory response more effectively than UC-MSCs.

### 3.3. Antifibrotic Effects of UC-MSCs and DPSCs on BEAS-2B and MRC-5 Cells

**Figure 3:**
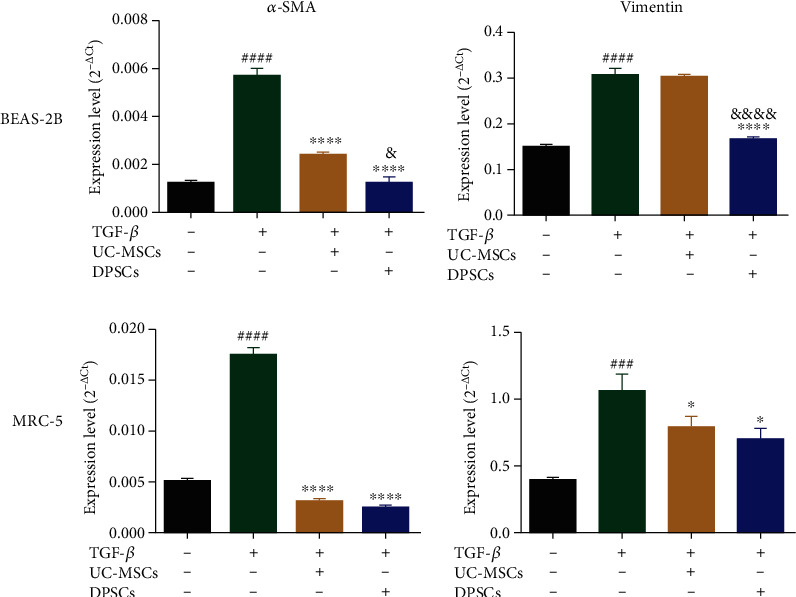
DPSCs alleviate the fibrotic response in BEAS-2B and MRC-5 cells better than UC-MSCs. The expression of the fibrosis-related cytokines *α*-SMA and vimentin in BEAS-2B and MRC-5 cells stimulated with TGF-*β* was evaluated by qPCR. Each experiment was performed in triplicate; the data are shown as the means ± SEM. ANOVA followed by Tukey's multiple comparisons test was used to evaluate these data. ^###^*P* < 0.001 and ^####^*P* < 0.0001 vs. Con; ^∗^*P* < 0.05 and ^∗∗∗∗^*P* < 0.0001 vs. TGF-*β*; ^&^*P* < 0.05 and ^&&&&^*P* < 0.0001 vs. UC-MSCs. Con: control; UC-MSCs: umbilical cord mesenchymal stem cells; DPSCs: dental pulp mesenchymal stem cells; *α*-SMA: *α*-smooth muscle actin.

*α*-Smooth muscle actin (*α*-SMA) and vimentin (VIM) were analyzed in both BEAS-2B and MRC-5 cells to examine the differences of the antifibrotic effects of the two MSC groups. *α*-SMA and VIM increased significantly in the TGF-*β* group, showing that BEAS-2B and MRC-5 underwent fibrosis (Figure 3). After MSCs administration, the expression levels of *α*-SMA and VIM in both cell lines were decreased to different degrees, especially *α*-SMA, which was substantially decreased. There was no statistically significant difference in the expression level of VIM in BEAS-2B cells between the UC-MSC group and the TGF-*β* group, while VIM was notably downregulated in the DPSCs group. These results indicated that DPSCs inhibited the expression of fibrotic factors more strongly than UC-MSCs. The antifibrotic effects of DPSCs-EVs were also analyzed, and the results suggested that EVs could decrease the expression of fibrotic factors in BEAS-2B cells, and the effect was better than that of supernatant of DPSCs (Supplementary Figure 1).

### 3.4. DPSCs-HGF Efficiently Alleviated BEAS-2B and MRC-5 Cell Inflammation

**Figure 4:**
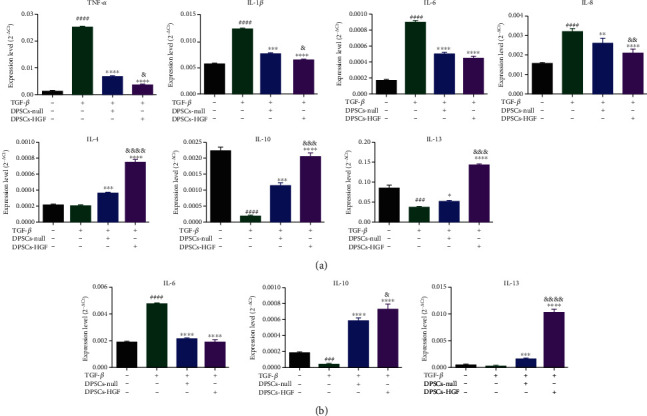
DPSCs-HGF inhibit the inflammatory response in BEAS-2B and MRC-5 cells. (a) The expression of inflammatory cytokines, including TNF-*α*, IL-1*β*, IL-6, IL-8, IL-4, IL-10, and IL-13, in BEAS-2B cells was measured by qPCR. (b) Inflammatory-related cytokines, including IL-6, IL-10, and IL-13, in MRC-5 cells were measured by qPCR. Each experiment was performed in triplicate. The data are shown as the means ± SEM. ANOVA followed by Tukey's multiple comparisons test was used to evaluate these data. ^###^*P* < 0.001 and ^####^*P* < 0.0001 vs. Con; ^∗^*P* < 0.05, ^∗∗^*P* < 0.01, ^∗∗∗^*P* < 0.001, and ^∗∗∗∗^*P* < 0.0001 vs. TGF-*β*; ^&^*P* < 0.05, ^&&^*P* < 0.01, ^&&&^*P* < 0.001, and ^&&&&^*P* < 0.0001 vs. DPSCs-null. Con: control; DPSCs: dental pulp mesenchymal stem cells.

Based on the previous results, we found that DPSCs had better anti-inflammatory and antifibrotic effects, and so, we modified DPSCs with the HGF gene and investigated the effect of DPSCs-HGF on the alleviation of inflammation and fibrosis. The results showed that the expression of TNF-*α*, IL-1*β*, IL-6, and IL-8 was decreased and IL-4, IL-10, and IL-13 were increased to varying degrees in BEAS-2B cells after DPSCs supernatant treatment (Figure 4(a)). Compared to the DPSCs-null group, DPSCs-HGF markedly affected the expression of TNF-*α*, IL-1*β*, IL-8, IL-4, IL-10, and IL-13. In MRC-5 cells, consistent with BEAS-2B cells, the two DPSCs groups exhibited suppressed IL-6 but increased IL-10 and IL-13 expression (Figure 4(b)). The expression of IL-13 was statistically significant in the DPSCs-HGF group compared with the DPSCs-null group. These results indicated that HGF modification could strengthen the anti-inflammatory ability of DPSCs.

### 3.5. DPSCs-HGF Inhibited Fibrosis in BEAS-2B and MRC-5 Cells

**Figure 5:**
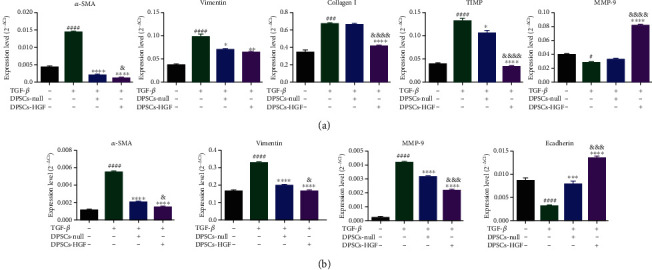
DPSCs-HGFs inhibit the fibrotic response in BEAS-2B and MRC-5 cells. (a) The expression of fibrotic factors, including *α*-SMA, vimentin, Col I, TIMP, and MMP-9, in MRC-5 cells stimulated with TGF-*β* was measured by qPCR. (b) The expression of fibrotic factors, including *α*-SMA, VIM, E-cadherin, and MMP-9, in BEAS-2B cells stimulated with TGF-*β* was measured by qPCR. Each experiment was performed in triplicate; the data are shown as the means ± SEM. ANOVA followed by Tukey's multiple comparisons test was used to evaluate these data. ^#^*P* < 0.05, ^###^*P* < 0.001, and ^####^*P* < 0.0001 vs. Con; ^∗^*P* < 0.05, ^∗∗^*P* < 0.01, ^∗∗∗^*P* < 0.001, and ^∗∗∗∗^*P* < 0.0001 vs. TGF-*β*; ^&^*P* < 0.05, ^&&&^*P* < 0.001, and ^&&&&^*P* < 0.0001 vs. DPSCs-null. Con: control; DPSCs: dental pulp mesenchymal stem cells; *α*-SMA: *α*-smooth muscle actin; Col I: collagen type I; TIMP: tissue inhibitor of metalloproteinase; MMP-9: matrix metalloproteinase 9.

To investigate the antifibrotic effects of DPSCs-HGF, fibrosis factors such as collagen I, TIMP, and MMP-9 were analyzed. The results showed that the expression of *α*-SMA, VIM, collagen I, and TIMP in MRC-5 cells was increased in the TGF-*β* group, suggesting that MRC-5 cells underwent fibrosis (Figure 5(a)). These fibrosis-related factors were decreased in the two DPSCs groups, particularly *α*-SMA. Compared to the DPSCs-null group, the DPSCs-HGF group exhibited decreased *α*-SMA, collagen I, and TIMP. TGF-*β* decreased MMP-9 in MRC-5 cells, but DPSCs-HGF increased MMP-9. These results indicated that DPSCs-HGF could enhance the fibrosis inhibition effect of DPSCs on MRC-5 cells.

*α*-SMA, VIM, MMP-9, and E-cadherin were also measured in BEAS-2B cells (Figure 5(b)). We found that DPSCs-HGF decreased *α*-SMA, VIM, and MMP-9 and increased E-cadherin considerably compared to those of DPSCs-null. These results suggested that DPSCs-HGF could inhibit the fibrosis of BEAS-2B cell and performed better than that of DPSCs-null.

### 3.6. DPSCs-HGF Protected against Lung Injury Induced by PQ In Vivo

**Figure 6:**
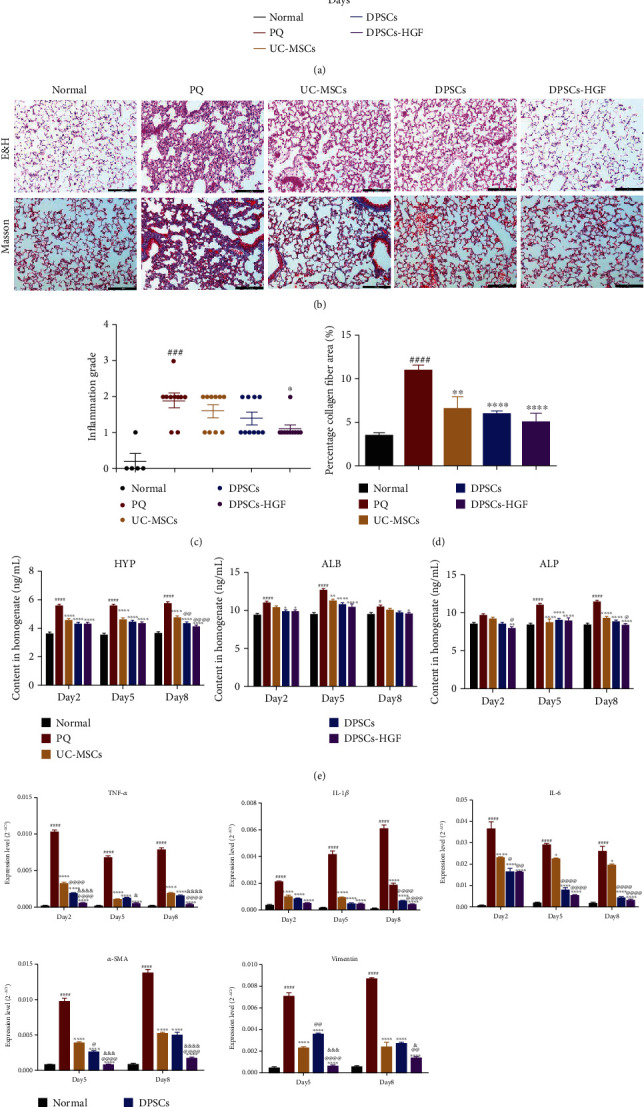
DPSCs-HGF transplantation protects against PQ-induced lung injury. (a) Survival rates were analyzed on day 28. (b) H&E staining and Masson staining were performed on lung sections. The nucleus was stained red by H&E, and collagen fibers were stained blue by Masson's trichrome. (c) The inflammation grade was analyzed. ANOVA followed by Dunn's multiple comparisons test was used to evaluate these data. (d) The percentage of the collagen fiber area was analyzed by Image-Pro Plus 6.0 software, and 10 random visual fields at 400x magnification were selected from each slice. (e) The expression of HYP, ALB, and ALP in BALF was detected by ELISA. (f) The expression of inflammation-related and fibrosis-related factors, including TNF-*α*, IL-1*β*, IL-6, *α*-SMA, and vimentin, was analyzed by qPCR. Each experiment was performed in triplicate; the data are shown as the means ± SEM. ANOVA followed by Tukey's multiple comparisons test was used to evaluate these data. ^#^*P* < 0.05, ^###^*P* < 0.001, and ^####^*P* < 0.0001 vs. normal; ^∗^*P* < 0.05, ^∗∗^*P* < 0.01, and ^∗∗∗∗^*P* < 0.0001 vs. PQ; ^@^*P* < 0.05, ^@@^*P* < 0.01, and ^@@@@^*P* < 0.0001 vs. UC-MSCs; ^&^*P* < 0.05, ^&&&^*P* < 0.001, and ^&&&&^*P* < 0.0001 vs. DPSCs. PQ: paraquat; H&E: hematoxylin and eosin; HYP: hydroxyproline; ALB: albumin; ALP: alkaline phosphatase; *α*-SMA: *α*-smooth muscle actin.

The survival curve was analyzed on day 28 after PQ gavage (Figure 6(a)). The survival rates of the control group, the PQ group, the UC-MSCs group, the DPSCs group, and the DPSCs-HGF group were 100%, 20%, 30%, 30%, and 50%, respectively. There was no significant difference in the UC-MSCs and DPSCs groups compared to the PQ group. However, the DPSCs-HGF group exhibited significantly increased survival rates compared to those of the PQ groups. These results indicated that DPSCs-HGF reduced the mortality of PQ-poisoned mice.

H&E staining was performed to investigate the degree of lung inflammation (Figures 6(b) and 6(c)). The results showed that the lung tissues of the normal group had structural integrity in the alveoli, without inflammatory infiltration. PQ induced alveolar structural damage, pulmonary interstitial congestion, and edema, as well as apparent infiltration of inflammatory cells. UC-MSCs and DPSCs treatment alleviated lung injury, but perivascular inflammation and pleurisy still occurred. DPSCs-HGF inhibited inflammatory mediators, as the alveolar structure was almost integrated without hemorrhage or inflammatory cell infiltration.

Masson staining was performed to evaluate the progression of lung fibrosis (Figures 6(b) and 6(d)). The results showed hemorrhage and collapse, and the large areas stained blue represented collagen fiber accumulation in the PQ group. The three MSCs groups exhibited decreased blue areas, and the collagen area was greatly reduced in the DPSCs-HGF group. There was no marked thickening of alveolar walls in the DPSCs-HGF group. These results indicated that DPSCs-HGF effectively prevented fibrosis development and collagen accumulation.

The expression of ALB, ALP, and HYP in BALF was measured by ELISA to evaluate the lung permeability (Figure 6(e)). The results showed that UC-MSCs, DPSCs, and DPSCs-HGF all decreased the expression of HYP on day 2, day 5, and day 8; the ALP on day 5 and day 8; and the ALB on day 5. There was a decline in the expression of ALB on day 8 only in the DPSCs-HGF group. The two DPSCs groups decreased the expression of HYP on day 8 with significant differences, compared to the UC-MSCs group. This result illustrated that MSCs groups could mitigate lung permeability and DPSCs better than UC-MSCs.

Several inflammation-related and fibrosis-related factors in lung tissue were also detected by qPCR to confirm the effect of MSCs on PQ mice (Figure 6(f)). The data showed that three MSCs groups all decreased the expression level of TNF-*α*, IL-1*β*, and IL-6. The expression level of these inflammatory cytokines in the DPSCs and DPSCs-HGF groups has a significant difference when compared with the UC-MSCs group. Further, DPSCs-HGF performed better than DPSCs. The expression of *α*-SMA and vimentin was all decreased significantly by the three MSCs groups, and the HGF modification group seemed stronger than the other two groups.

## 4. Discussion

In the clinic, the primary methods for treating PQ are hemodialysis, blood filtration to remove toxins, and drugs, such as adiponectin, sodium ferulate combined with oxymatrine, and pirfenidone plus prednisolone [3, 24, 25]. However, these currently available treatments are ineffective in halting the progression of the disease, and the survival rates are still low. With low immunogenicity and powerful immunosuppressive and anti-inflammatory properties, MSCs have been used in many studies of several respiratory diseases, such as acute respiratory distress syndrome (ARDS), acute lung injury (ALI), and chronic obstructive pulmonary disease (COPD), and in clinical trials [26–29]. This suggests that MSCs transplantation may be an effective strategy to treat PQ poisoning.

To date, MSCs used to treat PQ poisoning have been usually derived from bone marrow [30–32]. One factor that affects the therapeutic effect of MSCs is their source. Previous studies have compared the effect of some different sources of MSCs on ARDS. Ren et al. investigated the effect of UC-MSCs and BM-MSCs on ALI and found that UC-MSCs secreted higher levels of anti-inflammatory cytokines compared with BM-MSCs [33]. Silva et al. found that BM-MSCs and AD-MSCs yielded greater beneficial effects than lung tissue mesenchymal stem cells on ARDS [34]. This showed that different sources of MSCs may result in diverse effects on respiratory diseases. However, there is no information regarding the optimal source of MSCs to treat PQ poisoning.

Therefore, we compared the anti-inflammatory and antifibrotic properties of UC-MSCs and DPSCs to find a suitable source of MSCs. We found that DPSCs possessed much stronger anti-inflammatory and antifibrosis abilities than UC-MSCs. Additionally, the number of cytokines secreted by MSCs seemed to be insufficient to prevent inflammation and fibrosis progression. Some researchers have used MSCs genetically modified with factors such as CXCR4, basic fibrosis growth factor (bFGF), and HGF to mitigate the effects of acute and chronic lung injuries and pulmonary fibrosis [35–37]. In this study, we introduced the HGF gene, and we observed that HGF-modified DPSCs could promote cell survival and suppress inflammation. The results suggested that the combined application of HGF gene therapy and MSCs could improve the therapeutic effect. Therefore, in this study, we modified MSCs from a suitable source with HGF to study its protective effect on PQ-induced lung injury.

A symptom of PQ poisoning in the lung is an inflammatory reaction in the early stage, and persistent inflammation can promote excessive proliferation of lung fibroblasts, accelerating fibroblast differentiation to myofibroblasts, leading to intra-alveolar and interstitial fibrosis in the late stage [38]. Therefore, we used human lung epithelial cells BEAS-2B and human lung fibroblasts MRC-5 and investigated the anti-inflammatory and antifibrotic effects of UC-MSCs and DPSCs in this study.

Previous studies have shown that MSCs are an excellent source of cells to initiate pulmonary cell proliferation [39]. Our results showed that both UC-MSCs and DPSCs engraftment could promote BEAS-2B cell proliferation and advance lung repair regardless of the source; however, UC-MSCs performed better than DPSCs (Figure 1). Ren et al. showed that UC-MSCs had higher cell proliferation abilities than DPSCs because of the secretion of more growth factors and more energy metabolism [12].

PQ can induce the release of several inflammatory cytokines. Wang et al. reported that PQ could induce the release of TNF-*α*, IL-1*β*, IL-6, and IL-8, leading to acute lung injury [40]. Lee et al. indicated that TNF-*α* was produced by alveolar macrophages during the early stage of lung injury and in the later fibrosis stage, triggering the release of various proinflammatory cytokines and leading to fibroblast proliferation [41]. We found that MSCs could suppress the expression of proinflammatory factors, such as TNF-*α*, IL-1*β*, and IL-6, and increase anti-inflammatory factors, such as IL-13, thus relieving the inflammatory effect of BEAS-2B and MRC-5 cells (Figure 2). This finding was consistent with those of some studies [42, 43]. IL-10 plays an important anti-inflammatory role by inhibiting inflammatory factors, including IL-6, TNF-*α*, and IL-1, to protect damaged tissues and organs in certain chronic inflammatory diseases [44]. Recently, Yamamoto et al. reported that IL-10 could significantly reduce collagen 1 and fiber connections stimulated by TGF-*β* [45]. In our study, the expression level of IL-10 in MRC-5 cells was increased significantly by DPSCs and DPSCs-HGF (Figures 2 and 4). These results illustrated that MSCs might inhibit inflammation as well as fibrosis by promoting IL-10 expression.

There were small differences between UC-MSCs and DPSCs. Lower levels of proinflammatory cytokines and higher levels of anti-inflammatory cytokines were secreted when BEAS-2B or MRC-5 cells were cultured with DPSCs' supernatant then UC-MSCs' supernatant. Additionally, the inhibition of proinflammatory cytokines and acceleration of anti-inflammatory factors were significantly enhanced after modification with HGF (Figure 4). Therefore, DPSCs, especially DPSCs-HGF, could prevent inflammation progression by suppressing TNF-*α* and promoting IL-10. This may be one of the mechanisms by which MSCs treat PQ-induced lung injury.

Fibrosis is a symptom in the later stage of PQ poisoning. Many researchers have demonstrated that the administration of MSCs can protect against and reverse pulmonary fibrosis [46, 47]. *α*-SMA is regarded as a biomarker of fibroblasts, and vimentin is an intermediate product during myofibroblast differentiation. We found that MSCs decreased *α*-SMA and vimentin, and DPSCs had a stronger effect than UC-MSCs (Figure 3). These results suggested that DPSCs might be an ideal source for preventing fibrosis.

Then, additional fibrotic factors, such as collagen I, TIMP, and MMP-9, were analyzed to evaluate the inhibitory effect of DPSCs-HGF on lung fibrosis progression. Myofibroblasts are considered a primary causative cell type in the progression of pulmonary fibrosis [48]. Myofibroblasts can be derived from fibroblast transdifferentiation [49, 50] and epithelial-mesenchymal transformation (EMT) [51, 52]. EMT participates in PQ-induced pulmonary fibrosis [53, 54]. During the EMT process, E-cadherin, an epithelial cell marker, is decreased, and *α*-SMA and vimentin are increased [55, 56]. We found that DPSCs-HGF increased E-cadherin in BEAS-2B cells (Figure 5). This result suggested that EMT could be inhibited by DPSCs-HGF.

The MMP family plays different roles in different cell types. The role of MMP-9 in fibroblasts involves the degradation of the extracellular matrix to inhibit fibrosis [57]. However, MMP-9 promotes epithelial cell fibrosis in the late stage [58]. This finding was consistent with our results showing that MMP-9 was indeed increased in MRC-5 cells and decreased in BEAS-2B cells by DPSCs-HGF. Therefore, these results provided insight into the mechanism by which DPSCs-HGF inhibit fibrosis through the secretion of various factors.

In addition, we found that DPSCs-EVs exhibited a stronger ability to inhibit inflammation and fibrosis than DPSCs' supernatant (Supplementary Figure 1). DPSCs-EVs have the advantages of small size and strong penetration. Currently, EVs have become a research hotspot because they do not exist in the safety consideration of cell therapy. The underlying mechanisms and effects of EVs need further investigation.

We established an animal model of PQ poisoning through intragastric administration of 150 mg/kg PQ. The survival rate in the UC-MSCs and DPSCs transplantation groups was only slightly higher than that in the PQ group (Figure 6(a)), which was not as good as we expected. There were two possible reasons for this effect. First, the mice underwent PQ gastric perfusion at a high dose to cause a 90% fatality rate according to our previous preexperiment results. Liu et al. established a PQ poisoning model by administering 25 mg/kg PQ by gavage [59]. Zhang et al. reported that BMSC therapy can protect against PQ-induced ALI in rats administered 20 mg/kg PQ by intraperitoneal injection [31]. Schapochnik et al. established lung fibrosis in C57/BL6 mice by a single injection of PQ (10 mg/kg; i.p.) [60]. Apparently, the dose of PQ in our study was significantly higher than that in other studies. Second, PQ poisoning patients usually receive medical treatments such as an emetic and gastric lavage for the first time, but these measures could not be applied to mice. Therefore, PQ was retained and absorbed more in the mice and induced more serious injury in the lung. This might be the main reason why the survival rate and histopathology in the UC-MSCs and DPSCs groups were not satisfactory. However, DPSCs-HGF transplantation improved the survival rate of PQ-poisoned mice from 20% to 50% (Figure 6(a)). DPSCs-HGF significantly reduced inflammatory cell infiltration and collagen fiber accumulation in the lungs (Figure 6(b)). The results of BALF illustrated that DPSCs-HGF improved the lung permeability; alongside, the expression of inflammatory and fibrosis-related cytokines of lung tissue also confirmed the stronger immune regulation effect of DPSCs-HGF (Figures 6(e) and 6(f)). The data in vivo was in accordance with that in vitro. Some researchers have reported that DPSCs have robust immunoregulatory effects [61, 62]. In addition, HGF, with its antifibrotic effect, has been applied in many lung injury diseases [63]. These results suggest that the combination of DPSCs and HGF gene therapy provides a promising new and effective therapeutic approach to protect the lung from PQ.

The safety of MSCs transplantation therapies in the clinic is always the focus concerned by researchers. To date, published clinical trial data have attested the safety and feasibility of MSCs in diverse diseases, ARDS inclusive [64]. Zheng et al. demonstrated that a low dose of 1 million adipose-derived hMSCs/kg was well tolerated in a phase 1b trial [65]. Two clinical trials phase 1 (NCT01775774) and phase 2a (NCT02097641) which were initiated by Matthay et al. have proved that the BM-MSCs were well tolerated in patients with moderate to severe ARDS [66, 67]. Thus, MSCs could serve as a safe cell source in regenerative medicine.

Therefore, our study evaluated the comprehensive therapeutic effects of UC-MSCs and DPSCs on acute lung injury induced by PQ for the first time. We found that DPSCs had a stronger ability to inhibit inflammation and fibrosis in BEAS-2B and MRC-5 cells than UC-MSCs. In addition, EVs from MSCs were superior to MSCs' supernatant. DPSCs-HGF could improve the survival status and histopathology of PQ mice by suppressing proinflammatory and profibrotic cytokines and promoting anti-inflammatory and antifibrotic cytokines in lung tissue cells, which may be a possible mechanism of the attenuation of the lung injury induced by PQ.

## 5. Conclusion

In this study, we provide a novel cell therapy involving DPSCs-HGF transfusion to treat PQ-induced lung injury. DPSCs-HGF have a robust ability to modulate inflammatory and fibrotic cytokines and inhibit lung inflammatory and pulmonary fibrosis. The paracrine mechanisms may explain their therapeutic and preventive effects. This is the first report showing that DPSCs are a suitable cell source and that the transplantation of DPSCs-HGF may be an effective approach to prevent the progression of lung inflammation and pulmonary fibrosis. These findings suggest a novel strategy for treating PQ poisoning patients.

## Figures and Tables

**Table 1:** The primer sequence for qRT-PCR.

Primer name	Forward primer sequence	Reverse primer sequence
GAPDH-human	GACAACTTTGGTATCGTGGA	AGGCAGGGATGATGTTCTGG
TNF-*α*-human	CCCTCCTTCAGACACCCT	GGTTGCCAGCACTTCACT
IL-1*β*-human	TTGAGTCTGCCCAGTTCC	TTTCTGCTTGAGAGGTGCT
IL-4-human	GCACCGAGTTGACCGTA	GCAGCGAGTGTCCTTCTC
IL-6-human	CAATAACCACCCCTGACC	GCGCAGAATGAGATGAGTT
IL-8-human	GTGCTGTGTTGAATTACGGA	TTGACTGTGGAGTTTTGGC
IL-10-human	ACCAAGACCCAGACATCAA	CATTCTTCACCTGCTCCAC
IL-13-human	TAGCCGACCTCAGCCTT	TGCCTGTGTGTGAAGTGG
*α*-SMA-human	CTATGCCTCTGGACGCACAAC	CCCATCAGGCAACTCGTAACTC
Vimentin-human	TCCGCACATTCGAGCAAAGA	TGATTCAAGTCTCAGCGGGC
MMP-9-human	ACGCAGACATCGTCATCC	CCAGGGACCACAACTCG
TIMP-human	TTCCAGTCCCGTCACCTT	CAGGCTTCAGCTTCCACTC
E-cadherin-human	GGTTGATCCTGGCTTTGTT	GCCCTGTTGTCCTTCTTTT
Collagen I-human	AGACGAAGACATCCCACCA	GTCGCAGACGCAGATCC
*β*-Actin-mouse	CCTCACTGTCCACCTTCC	GGGTGTAAAACGCAGCTC
*α*-SMA-mouse	GCCCAGAGCAAGAGAGG	TGTCAGCAGTGTCGGATG
Vimentin-mouse	CTCCTACGATTCACAGCCA	GAGCCACCGAACATCCT
TNF-*α*-mouse	CGCTGAGGTCAATCTGC	GGCTGGGTAGAGAATGGA
IL-1*β*-mouse	CCAAGCTTCCTTGTGCAAGTA	AAGCCCAAAGTCCATCAGTGG
IL-6-mouse	ACAGAAGGAGTGGCTAAGGA	AGGCATAACGCACTAGGTTT

## Data Availability

All data generated and/or analyzed during this study are included in this published article. Data sharing is not applicable to this article as no datasets were generated or analyzed during the current study. However, the data that support the findings of this study are available from the corresponding authors upon reasonable request.

## References

[1] Dinis-Oliveira R. J., Duarte J. A., Sanchez-Navarro A., Remiao F., Bastos M. L., Carvalho F. (2008). Paraquat poisonings: mechanisms of lung toxicity, clinical features, and treatment. *Critical Reviews in Toxicology*.

[2] Wesseling C., van Wendel de Joode B., Ruepert C. (2001). Paraquat in developing countries. *International Journal of Occupational and Environmental Health*.

[3] Gawarammana I. B., Buckley N. A. (2011). Medical management of paraquat ingestion. *British Journal of Clinical Pharmacology*.

[4] Fan H., Huang H., Hu L. (2018). The activation of STIM1 mediates S-phase arrest and cell death in paraquat induced acute lung intoxication. *Toxicology Letters*.

[5] Gunawardena G., Roberts D. M., Buckley N. A. (2007). Randomized control trial of immunosuppression in paraquat poisoning. *Critical Care Medicine*.

[6] Gawarammana I. B., Dawson A. H. (2010). Peripheral burning sensation: a novel clinical marker of poor prognosis and higher plasma-paraquat concentrations in paraquat poisoning. *Clinical Toxicology (Philadelphia, Pa.)*.

[7] Lohitnavy M., Chitsakhon A., Jomprasert K., Lohitnavy O., Reisfeld B. Development of a physiologically based pharmacokinetic model of paraquat.

[8] Crisan M., Yap S., Casteilla L. (2008). A perivascular origin for mesenchymal stem cells in multiple human organs. *Cell Stem Cell*.

[9] Aggarwal S., Pittenger M. F. (2005). Human mesenchymal stem cells modulate allogeneic immune cell responses. *Blood*.

[10] Hoffmann A., Floerkemeier T., Melzer C., Hass R. (2017). Comparison of in vitro-cultivation of human mesenchymal stroma / stem cells derived from bone marrow and umbilical cord. *Journal of Tissue Engineering and Regenerative Medicine*.

[11] Liao H. T., Chen C. T. (2014). Osteogenic potential: comparison between bone marrow and adipose-derived mesenchymal stem cells. *World Journal of Stem Cells*.

[12] Ren H., Sang Y., Zhang F., Liu Z., Qi N., Chen Y. (2016). Comparative analysis of human mesenchymal stem cells from umbilical cord, dental pulp, and menstrual blood as sources for cell therapy. *Stem Cells International*.

[13] Molnarfi N., Benkhoucha M., Funakoshi H., Nakamura T., Lalive P. H. (2015). Hepatocyte growth factor: a regulator of inflammation and autoimmunity. *Autoimmunity Reviews*.

[14] Meng F., Meliton A., Moldobaeva N. (2015). Asef mediates HGF protective effects against LPS-induced lung injury and endothelial barrier dysfunction. *American Journal of Physiology. Lung Cellular and Molecular Physiology*.

[15] Hu S., Li J., Xu X. (2016). The hepatocyte growth factor-expressing character is required for mesenchymal stem cells to protect the lung injured by lipopolysaccharide in vivo. *Stem Cell Research & Therapy*.

[16] Shukla M. N., Rose J. L., Ray R., Lathrop K. L., Ray A., Ray P. (2009). Hepatocyte growth factor inhibits epithelial to myofibroblast transition in lung cells via Smad7. *American Journal of Respiratory Cell and Molecular Biology*.

[17] Watanabe M., Ebina M., Orson F. M. (2005). Hepatocyte growth factor gene transfer to alveolar septa for effective suppression of lung fibrosis. *Molecular Therapy*.

[18] Witwer K. W., Buzás E. I., Bemis L. T. (2013). Standardization of sample collection, isolation and analysis methods in extracellular vesicle research. *Journal of Extracellular Vesicles*.

[19] Théry C., Ostrowski M., Segura E. (2009). Membrane vesicles as conveyors of immune responses. *Nature Reviews. Immunology*.

[20] Li T., Xia M., Gao Y., Chen Y., Xu Y. (2015). Human umbilical cord mesenchymal stem cells: an overview of their potential in cell-based therapy. *Expert Opinion on Biological Therapy*.

[21] Wang H., Yang Y. F., Zhao L. (2013). Hepatocyte growth factor gene-modified mesenchymal stem cells reduce radiation-induced lung injury. *Human Gene Therapy*.

[22] Kong F., Wu C.-T., Geng P. (2020). Dental pulp stem cell-derived extracellular vesicles mitigate haematopoietic damage after radiation. *Stem Cell Reviews and Reports*.

[23] Murao Y., Loomis W., Wolf P., Hoyt D. B., Junger W. G. (2003). Effect of dose of hypertonic saline on its potential to prevent lung tissue damage in a mouse model of hemorrhagic shock. *Shock*.

[24] Yao R., Zhou Y., He Y. (2015). Adiponectin protects against paraquat-induced lung injury by attenuating oxidative/nitrative stress. *Experimental and Therapeutic Medicine*.

[25] Rasooli R., Pourgholamhosein F., Kamali Y., Nabipour F., Mandegary A. (2018). Combination therapy with pirfenidone plus prednisolone ameliorates paraquat-induced pulmonary fibrosis. *Inflammation*.

[26] Lopes-Pacheco M., Robba C., Rocco P. R. M., Pelosi P. (2020). Current understanding of the therapeutic benefits of mesenchymal stem cells in acute respiratory distress syndrome. *Cell Biology and Toxicology*.

[27] Qin H., Zhao A. (2020). Mesenchymal stem cell therapy for acute respiratory distress syndrome: from basic to clinics. *Protein & Cell*.

[28] Bich P. L. T., Thi H. N., Chau H. D. N. (2020). Allogeneic umbilical cord-derived mesenchymal stem cell transplantation for treating chronic obstructive pulmonary disease:a pilot clinical study. *Stem Cell Research & Therapy*.

[29] Weiss D. J., Casaburi R., Flannery R., LeRoux-Williams M., Tashkin D. P. (2013). A placebo-controlled, randomized trial of mesenchymal stem cells in COPD. *Chest*.

[30] He F., Feng A. Z. S., Li Y., Liu T. (2018). Mesenchymal stem cell therapy for paraquat poisoning: a systematic review and meta-analysis of preclinical studies. *PLoS One*.

[31] Zhang L., Li Q., Liu Z., Yu W., Zhao M. (2019). The protective effects of bone mesenchymal stem cells on paraquat-induced acute lung injury via the muc5b and ERK/MAPK signaling pathways. *American Journal of Translational Research*.

[32] Zhang L., Yu W., Shen H., Zhao M. (2020). Combined signaling of NF-kappaB and IL-17 contributes to mesenchymal stem cells-mediated protection for paraquat-induced acute lung injury 17 contributes to mesenchymal stem cells-mediated protection for paraquat-induced acute lung injury. *BMC Pulmonary Medicine*.

[33] Ren H., Zhang Q., Wang J., Pan R. (2018). Comparative effects of umbilical cord- and menstrual blood-derived MSCs in repairing acute lung injury. *Stem Cells International*.

[34] Silva J. D., Lopes-Pacheco M., Paz A. H. R. (2018). Mesenchymal stem cells from bone marrow, adipose tissue, and lung tissue differentially mitigate lung and distal organ damage in experimental acute respiratory distress syndrome. *Critical Care Medicine*.

[35] Yang J.-X., Zhang N., Wang H.-W., Gao P., Yang Q.-P., Wen Q.-P. (2015). CXCR4 Receptor Overexpression in Mesenchymal Stem Cells Facilitates Treatment of Acute Lung Injury in Rats. *The Journal of Biological Chemistry*.

[36] Zhao Y. F., Luo Y. M., Xiong W. (2015). Mesenchymal stem cell-based FGF2 gene therapy for acute lung injury induced by lipopolysaccharide in mice. *European Review for Medical and Pharmacological Sciences*.

[37] Chen S., Chen X., Wu X. (2017). Hepatocyte growth factor-modified mesenchymal stem cells improve ischemia/reperfusion-induced acute lung injury in rats. *Gene Therapy*.

[38] Toygar M., Aydin I., Agilli M. (2015). The relation between oxidative stress, inflammation, and neopterin in the paraquat-induced lung toxicity. *Human & Experimental Toxicology*.

[39] Karaoz E., Genc Z. S., Demircan P. C., Aksoy A., Duruksu G. (2010). Protection of rat pancreatic islet function and viability by coculture with rat bone marrow-derived mesenchymal stem cells. *Cell Death & Disease*.

[40] Wang X., Luo F., Zhao H. (2014). Paraquat-induced reactive oxygen species inhibit neutrophil apoptosis via a p38 MAPK/NF-*κ*B–IL-6/TNF-*α* positive-feedback circuit. *PLoS One*.

[41] Lee I. T., Lin C. C., Wu Y. C., Yang C. M. (2010). TNF-*α* induces matrix metalloproteinase-9 expression in A549 cells: role of TNFR1/TRAF2/PKC*α*-dependent signaling pathways. *Journal of Cellular Physiology*.

[42] Gupta N., Su X., Popov B., Lee J. W., Serikov V., Matthay M. A. (2007). Intrapulmonary delivery of bone marrow-derived mesenchymal stem cells improves survival and attenuates endotoxin-induced acute lung injury in mice. *Journal of Immunology*.

[43] Nemeth K., Leelahavanichkul A., Yuen P. S. (2009). Bone marrow stromal cells attenuate sepsis via prostaglandin E_2_-dependent reprogramming of host macrophages to increase their interleukin-10 production. *Nature Medicine*.

[44] Moore K. W., de Waal Malefyt R., Coffman R. L., O'Garra A. (2001). INTERLEUKIN-10AND THEINTERLEUKIN-10 receptor. *Annual Review of Immunology*.

[45] Yamamoto T., Eckes B., Krieg T. (2001). Effect of interleukin-10 on the gene expression of type I collagen, fibronectin, and decorin in human skin fibroblasts: differential regulation by transforming growth factor-*β* and monocyte chemoattractant protein-1. *Biochemical and Biophysical Research Communications*.

[46] Wang C., Zhu H., Sun Z. (2014). Inhibition of Wnt/beta-catenin signaling promotes epithelial differentiation of mesenchymal stem cells and repairs bleomycin-induced lung injury. *American Journal of Physiology. Cell Physiology*.

[47] Bitencourt C. S., Pereira P. A. T., Ramos S. G. (2011). Hyaluronidase recruits mesenchymal-like cells to the lung and ameliorates fibrosis. *Fibrogenesis Tissue Repair*.

[48] Phan S. H. (1996). Role of the myofibroblast in pulmonary fibrosis. *Kidney International. Supplement*.

[49] Selman M., Thannickal V. J., Pardo A., Zisman D. A., Martinez F. J., Lynch J. P. (2004). Idiopathic pulmonary fibrosis. *Drugs*.

[50] Thannickal V. J., Lee D. Y., White E. S. (2003). Myofibroblast Differentiation by Transforming Growth Factor-ॆ1 Is Dependent on Cell Adhesion and Integrin Signaling via Focal Adhesion Kinase. *Journal of Biological Chemistry*.

[51] Kasai H., Allen J. T., Mason R. M., Kamimura T., Zhang Z. (2005). TGF-beta1 induces human alveolar epithelial to mesenchymal cell transition (EMT). *Respiratory Research*.

[52] Willis B. C., Liebler J. M., Luby-Phelps K. (2005). Induction of epithelial-mesenchymal transition in alveolar epithelial cells by transforming growth factor-*β*1: potential role in idiopathic pulmonary fibrosis. *The American Journal of Pathology*.

[53] Zhu Y., Tan J., Xie H., Wang J., Meng X., Wang R. (2016). HIF‐1*α* regulatesEMTviathe Snail and *β*-catenin pathways in paraquat poisoning-induced early pulmonary fibrosis. *Journal of Cellular and Molecular Medicine*.

[54] Sun H., Chen J., Qian W. (2016). Integrated long non‐codingRNAanalyses identify novel regulators of epithelial-mesenchymal transition in the mouse model of pulmonary fibrosis. *Journal of Cellular and Molecular Medicine*.

[55] Peinado H., Olmeda D., Cano A. (2007). Snail, Zeb and bHLH factors in tumour progression: an alliance against the epithelial phenotype?. *Nature Reviews Cancer*.

[56] Sarkar T. R., Battula V. L., Werden S. J. (2015). GD3 synthase regulates epithelial-mesenchymal transition and metastasis in breast cancer. *Oncogene*.

[57] Bonnans C., Chou J., Werb Z. (2014). Remodelling the extracellular matrix in development and disease. *Nature Reviews Molecular Cell Biology*.

[58] Zhang F., Hu L., Wu Y.-x. (2019). Doxycycline alleviates paraquat-induced acute lung injury by inhibiting neutrophil-derived matrix metalloproteinase 9. *International Immunopharmacology*.

[59] Liu B., Chen A., Lan J., Ren L., Wei Y., Gao L. (2019). Protective mechanism of 1-methylhydantoin against lung injury induced by paraquat poisoning. *PLoS One*.

[60] Schapochnik A., da Silva M. R., Leal M. P. (2018). Vitamin D treatment abrogates the inflammatory response in paraquat-induced lung fibrosis. *Toxicology and Applied Pharmacology*.

[61] Sonoyama W., Liu Y., Yamaza T. (2008). Characterization of the apical papilla and its residing stem cells from human immature permanent teeth: a pilot study. *Journal of Endodontia*.

[62] Ishizaka R., Hayashi Y., Iohara K. (2013). Stimulation of angiogenesis, neurogenesis and regeneration by side population cells from dental pulp. *Biomaterials*.

[63] Chakraborty S., Chopra P., Hak A., Dastidar S. G., Ray A. (2013). Hepatocyte growth factor is an attractive target for the treatment of pulmonary fibrosis. *Expert Opinion on Investigational Drugs*.

[64] Walter J., Ware L. B., Matthay M. A. (2014). Mesenchymal stem cells: mechanisms of potential therapeutic benefit in ARDS and sepsis. *The Lancet Respiratory Medicine*.

[65] Zheng G., Huang L., Tong H. (2014). Treatment of acute respiratory distress syndrome with allogeneic adipose-derived mesenchymal stem cells: a randomized, placebo-controlled pilot study. *Respiratory Research*.

[66] Wilson J. G., Liu K. D., Zhuo H. (2015). Mesenchymal stem (stromal) cells for treatment of ARDS: a phase 1 clinical trial. *The Lancet Respiratory Medicine*.

[67] Matthay M. A., Calfee C. S., Zhuo H. (2019). Treatment with allogeneic mesenchymal stromal cells for moderate to severe acute respiratory distress syndrome (START study): a randomised phase 2a safety trial. *The Lancet Respiratory Medicine*.

