# Supplementary material for: HGF-Modified Dental Pulp Stem Cells Mitigate the Inflammatory and Fibrotic Responses in Paraquat-Induced Acute Respiratory Distress Syndrome
Source: Stem Cells Int. 2021 Mar 2;2021:6662831. doi: 10.1155/2021/6662831 (PMC7943272; doi:10.1155/2021/6662831)
Supplement: Supplementary Materials — Data on DPSCs-EVs can be found in Supplementary Figure 1. [file 6662831.f1.docx]

**
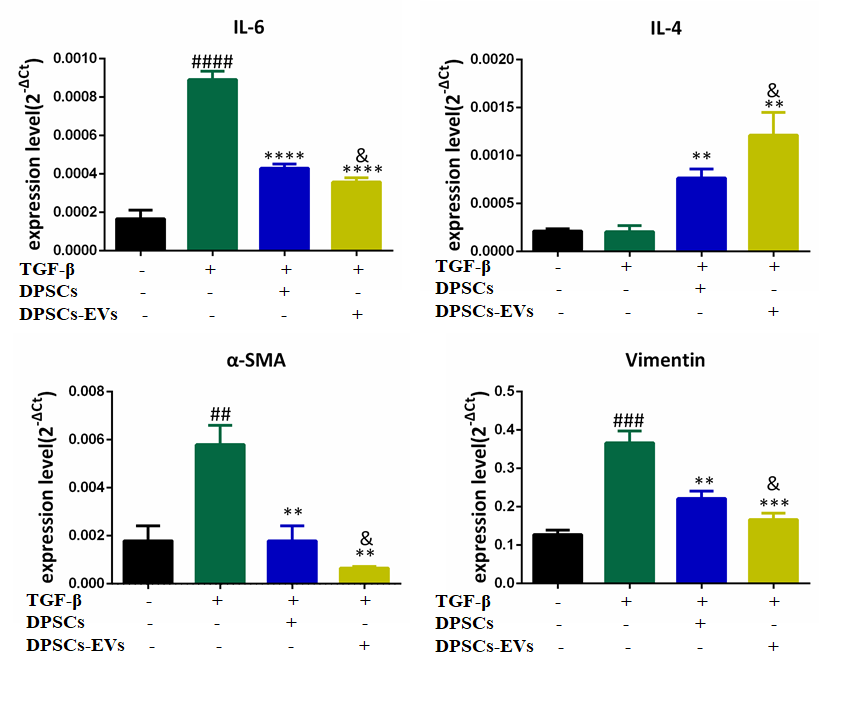
**

**Supplementary Figure 1.** DPSCs-EVs alleviate the inflammatory and fibrotic responses of BEAS-2B cells. The expression of the inflammatory cytokines IL-4 and IL-6 and the fibrosis-related cytokines α-SMA and Vimentin in BEAS-2B cells were evaluated by qPCR. Each experiment was performed in triplicate; the data are shown as the means ± SEM. ANOVA followed by Tukey’s multiple comparisons test was used to evaluate these data. ^##^P<0.01, ^###^P<0.001, ^####^P<0.0001 vs Con; **P<0.01, ***P<0.001，****P<0.0001 vs TGF-β; ^&^P<0.05 vs DPSCs. Con: control; DPSCs: dental pulp mesenchymal stem cells; α-SMA: α-smooth muscle actin.
